# Peroxisomal proliferator activated receptor-γ deficiency in a Canadian kindred with familial partial lipodystrophy type 3 (FPLD3)

**DOI:** 10.1186/1471-2350-7-3

**Published:** 2006-01-14

**Authors:** Gordon A Francis, Gang Li, Robin Casey, Jian Wang, Henian Cao, Todd Leff, Robert A Hegele

**Affiliations:** 1 Department of Medicine and Biochemistry, University of Alberta, Edmonton, Alberta, T6G 2S2, Canada; 2 Department of Pathology, Wayne State University School of Medicine, Detroit, Michigan, 48201, USA; 3 Department of Medical Genetics, University of Calgary, Calgary, Alberta, Canada T2T 5C7; 4 Robarts Research Institute, London, Ontario, N6A 5K8, Canada; 5 Department of Medicine, University of Western Ontario, London, Ontario, N6A 5K8, Canada

## Abstract

**Background:**

Familial partial lipodystrophy (Dunnigan) type 3 (FPLD3, Mendelian Inheritance in Man [MIM] 604367) results from heterozygous mutations in *PPARG *encoding peroxisomal proliferator-activated receptor-γ. Both dominant-negative and haploinsufficiency mechanisms have been suggested for this condition.

**Methods:**

We present a Canadian FPLD3 kindred with an affected mother who had loss of fat on arms and legs, but no increase in facial, neck, suprascapular or abdominal fat. She had profound insulin resistance, diabetes, severe hypertriglyceridemia and relapsing pancreatitis, while her pre-pubescent daughter had normal fat distribution but elevated plasma triglycerides and C-peptide and depressed high-density lipoprotein cholesterol.

**Results:**

The mother and daughter were each heterozygous for *PPARG *nonsense mutation Y355X, whose protein product *in vitro *was transcriptionally inactive with no dominant-negative activity against the wild-type receptor. In addition the mutant protein appeared to be markedly unstable.

**Conclusion:**

Taken together with previous studies of human *PPARG *mutations, these findings suggest that PPAR-γ deficiency due either to haploinsufficiency or to substantial activity loss due to dominant negative interference of the normal allele product's function can each contribute to the FPLD3 phenotype.

## Background

Dunnigan-type familial partial lipodystrophy (FPLD), a genetically heterogeneous condition with at least three subtypes, is considered to be a monogenic model of the common "metabolic syndrome" [1]. FPLD subtype 2 (FPLD2, MIM 151660) results from mutations in nuclear lamin A/C encoded by *LMNA *(MIM 151330), and FPLD subtype 3 (FPLD3, MIM 604367) results from mutations in peroxisome proliferator-activated receptor-γ encoded by *PPARG *(MIM 601487) [1]. The FPLD phenotype is characterized by redistribution of fat stores – lipoatrophy of extremities and gluteal region often with lipohypertrophy involving the face and in central/visceral adipose stores – together with clinical metabolic disturbances such as hyperlipidemia, hypertension and diabetes [2]. Careful phenotypic – or "phenomic" – analysis suggested that fat redistribution is more extreme in FPLD2 compared to FPLD3, but that metabolic disturbances are greater in FPLD3 compared to FPLD2 [1-3]. Thus, fat loss is probably a key mechanism that contributes to the development of metabolic complications in both FPLD subtypes, but the more severe clinical features in FPLD3 suggest that *PPARG *mutations may have additional direct effects. Indeed, an independent direct effect of the dominant negative human *PPARG *P467L mutation – induction of hypertension – was recently demonstrated in an animal model [4,5].

Certain *PPARG *missense mutations in FPLD3 – such as V290M and P467L – appear to act through a dominant negative mechanism, in which the mutant allele product is expressed and then interferes with the function of the product of the wild-type allele, resulting in markedly depressed net receptor activity [6,7]. In contrast, the heterozygous *PPARG *missense mutation F388L in FPLD3 does not act through a dominant negative mechanism; it appears simply to have diminished transactivation capacity, with no obvious effect on the wild-type allele product, resulting in effective "haploinsufficiency" from depressed but not absent receptor activity [8]. Furthermore, the recently described heterozygous *PPARG *-14A>G promoter variant in FPLD3 is associated with no coding sequence change, and only a simple reduction in promoter activity [9], again suggesting that haploinsufficiency but not complete deficiency of PPAR-γ activity as the basis of disease among affected heterozygotes. We now present a Canadian FPLD3 family with a novel *PPARG *nonsense mutation whose disease mechanism appears to be haploinsufficiency rather than dominant negative.

## Methods

### DNA sequence analysis

The study received approval from the University of Western Ontario Ethics Review Panel (protocol 07920E) and all subjects gave informed consent to participate. DNA sequencing initially showed no mutation in *LMNA *[4]. We amplified and sequenced the 6 exons of *PPARG *plus >100 nucleotides at each intron-exon boundary, and ~700 bp of the promoter. Rapid, allele-specific genotyping methods were then developed for each mutation. For the Y355X mutation genotype, we amplified the 603 bp fragment containing exon 5 using primers 5' TTC ACT GTG AGT TAG AAA TC and 3' CAA TGC AGA CTA ACA CTA AGG. This was followed by electrophoresis in 2% agarose, gel purification and ddNTP extension (SnaPShot, PE Applied Biosystems, Mississauga, ON) with primer 5' AAG AGC CTG CGA AAG CCT TT, and analysis on a Prism 377 DNA Sequencer (PE Applied Biosystems, Mississauga, ON). Genomic DNA from 260 healthy Caucasian subjects was studied, permitting 95% power to exclude a mutation frequency ≥1% in the healthy population (two-tailed alpha<0.05).

### PPARγ clones and transcription assays

A cDNA encoding full-length human PPARγ1 was cloned into the eukaryotic expression vector pcDNA4/HisMax-TOPO (Invitrogen, Carlsbad, CA). The Y355X mutation was introduced into this clone using the Quick-Change mutagenesis kit (Stratagene, La Jolla, CA) by changing the tyrosine codon (TAC) to a stop codon TAG. Both the wild-type (WT) and mutant clones were fully sequenced. Initial experiments demonstrated that transfection of the Y355X PPARγ clone resulted in the production of a small amount of full-length PPARγ protein, presumably generated by inefficient translation termination at the 355X stop codon. To eliminate this contaminating wild-type PPARγ, which would compromise interpretation of results, a second construct was generated in which all of the PPARγ sequences down-stream of the 355X codon were deleted from the plasmid. All of the transcription assays were carried out with this construct. For transcription assays, NIH3T3 mouse fibroblasts were grown in 96-well plates (5.5 × 10^3 ^cells/well) in DMEM + 10% fetal calf serum. Cells were transfected with the either WT or Y355X mutant PPARγ expression plasmid, 1 ng of a β-galactosidase control plasmid and 35 ng of the PPAR-dependent luciferase reporter pFATP-Luc (three copies of the mouse FATP gene PPRE inserted upstream of the minimal thymidine kinase promoter). Cells were transfected for 4 h with Lipofectamine-plus (Invitrogen, Carlsbad, CA) and then treated with DMSO or the indicated amount of rosiglitazone for 16 h. Luciferase and β-galactosidase activities were measured in cell extracts using the Dual-light assay system (ABI, Foster City, CA) and a 96-well luminometer (Berthold Technologies, Bad Wildbad, Germany). Transfections were performed in triplicate.

### Western analysis of wild-type and mutant PPARγ

To examine protein expression of PPARγ, a FLAG epitope tag was added to the N-termini of WT and Y355X cDNAs and both were transferred into the eukaryotic expression vector pTRE-shuttle2 (BD Biosciences Clontech, Palo Alto, CA). NIH3T3 cells (1.0 × 10^6 ^cells/well of a 6-well plate) were transfected with the indicated amounts of PPARγ expression plasmid, and 1 ng of a cyan fluorescent protein-expressing plasmid pECFP-N1 as a control. After 20 h, cells were washed once in phosphate-buffered saline (PBS) and then lysed in a buffer supplemented with 1% Triton X-100, 50 mM HEPES, 150 mM NaCl, 1 mM EDTA, 30 mM NaF and 1 mM Na_3_VO_4_. Protein concentration was determined by Bradford assay (Bio-Rad), and equal amounts of protein were analyzed by SDS gel electrophoresis. Western blots were performed by using monoclonal anti-FLAG-M2-peroxidase (HRP) conjugated antibody (Sigma, St. Louis, MO).

## Results

### Subjects' medical histories and clinical studies

**Figure 1:**
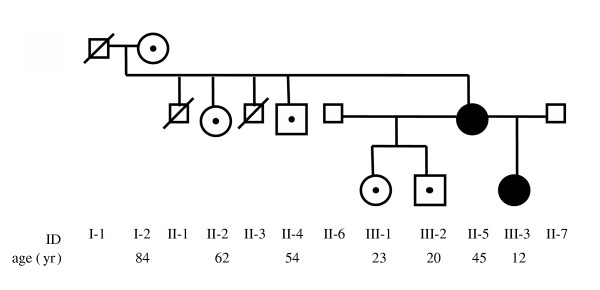
**A Canadian multigenerational FPLD3 kindred**. Darkened symbols indicate affected individuals, each of whom was later confirmed to be heterozygous for the respective *PPARG *nonsense mutation. Dots inside white symbols indicate normal *PPARG *genotype. Subject identification number and age in years are shown below the appropriate symbols.

**Figure 2:**
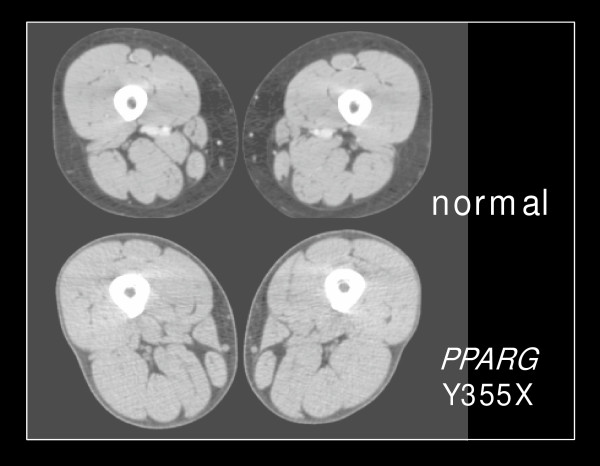
**Selected imaging studies from the FPLD3 subject**. Panel shows computed tomography cross-section of upper thigh of a normal individual and subject II-5, who had markedly decreased subcutaneous fat on both extremities.

*Subject II-5 *(Figure 1). A 45 year-old woman was referred for severe hypertriglyceridemia with relapsing pancreatitis. She had noted muscular legs and lower arms beginning in adolescence. Menarche was at age 12 and periods were regular. Type 2 diabetes mellitus and hypertriglyceridemia were diagnosed during her third pregnancy at age 33. Diabetes was treated with oral agents alone until age 40 when insulin was added. Control of both glycemia and hypertriglyceridemia has been difficult, with triglycerides >100 mmol/L at times and recurrent eruptive xanthomata and pancreatitis. A five-month trial of pioglitazone 15 mg daily resulted in a decrease in glycated haemoglobin (HbA1c) from 12.1 to 7.6%, and in triglycerides from 66.4 to 7.3 mmol/L, but this was later discontinued due to fluid retention. She had no history of hypertension. Physical examination revealed normal blood pressure, BMI 30 kg/m^2^, waist circumference 90 cm, and small eruptive xanthomata over her elbows. Subcutaneous fat was markedly diminished in her arms below the distal humerus and in her buttocks, thighs, and calves, with prominent arm and leg musculature. Facial, neck, suprascapular and abdominal fat distribution was normal. She had no buffalo hump, acanthosis nigricans or phlebectasia. Abdominal ultrasound showed an enlarged fatty liver and enlarged spleen. Dual X-ray absorptiometry scan revealed total body fat of 19.9% (within the normal female range), but with decreased fat on the limbs, particularly the legs at 14%. Figure 2 shows a computed tomographic scan of the legs of the proband and a normal subject; the proband had a relative paucity of fat. Measurements in fasting plasma included: glucose 14.4 mmol/L; HbA1c 10.1% (normal 4.3–6.1%); insulin 26.3 mU/L (normal 5.0–20.0); C-peptide 3.69 nmol/L (normal 0.30–1.32). Plasma concentrations of total cholesterol and triglycerides were 14.7 and 49.6 mmol/L; high-density lipoprotein (HDL) and low-density lipoprotein (LDL) cholesterol were not determined. Serum alanine transaminase was 21 U/L (normal <50 U/L). *APOE *genotype was normal (E3/E3). Lipoprotein lipase activity was slightly depressed at 79 nmol/min/ml (normal >90 nmol/min/ml). Triglycerides varied between 9.1 and 59.4 mmol/L despite treatment with fenofibrate 200 mg and salmon oil 6 g daily. Plasma glucose remained high despite taking >2 U/kg insulin daily and metformin 500 mg TID. A five-day trial of octreotide 100 μg TID to ameliorate abdominal pain reduced plasma C-peptide levels to <1 nmol/L and insulin to the normal range, but did not reduce fasting or postprandial glucose levels and produced abdominal bloating. Genomic DNA sequence analysis of the *LIPE *gene encoding lipoprotein lipase showed no mutations.

*Subject III-3 *(Figure 1). The 12 year-old daughter of subject II-5 was assessed for combined hyperlipidemia. She was born at 33 weeks' gestation, during a pregnancy that had been complicated by pre-eclampsia, diabetes and hypertriglyceridemia. At age 3 months, she was diagnosed with a patent ductus arteriosus with pulmonary branch stenosis and incomplete right bundle branch block. She had poor growth and difficulty feeding as an infant. She was first noted to have elevated triglycerides and cholesterol at age 5 that were treated only with dietary advice. She required several corrective surgeries for strabismus, the most recent at age 12. There was no history of pancreatitis or eruptive xanthomata. Height and weight at age 12 1/2 years were 153 cm (25–50^th ^percentile for age and sex) and 45.4 kg (50^th ^percentile for age and sex), respectively. She had a broad forehead with deep-set eyes, triangular facies and a narrow pointed chin. Examination of the chest, heart, and musculoskeletal systems was unremarkable, and there was no acanthosis nigricans or phlebectasia. She had a normal distribution of subcutaneous fat, with no loss of fat over the arms, buttocks, or legs, and no increase in face, neck, suprascapular or abdominal fat. Measurements in fasting plasma revealed: glucose 5.0 mmol/L; insulin 18.2 mU/L (normal 5.0–20 mU/L); C-peptide 1.64 nmol/L (normal 0.30–1.32 nmol/L); total cholesterol 5.26 mmol/L (normal 3.2–4.4 mmol/L for age), triglycerides 1.79 mmol/L (normal 0.4–1.3 mmol/L); HDL cholesterol 0.92 mmol/L (normal 1.0–1.8 mmol/L); and LDL cholesterol 3.52 (normal 1.6–2.8 mmol/L).

### DNA sequence analysis

**Figure 3:**
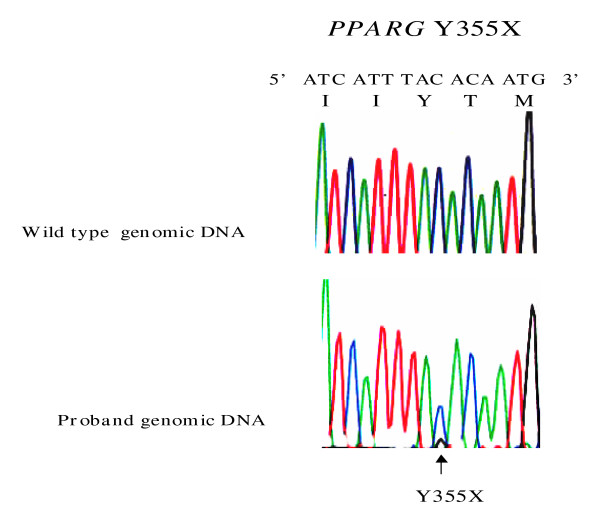
**Genomic DNA sequence electropherograms of heterozygous *PPARG *mutation**. The bottom electropherogram tracing shows both alleles in subject II-5 from kindred 1 (Y355X), compared to sequence from a healthy subject. The position of the mutation is indicated by the arrow. Normal nucleotide and amino acid sequence is shown above the wild type electropherogram tracing.

Subjects II-5 and III-3 were each found to be heterozygous for a nonsense mutation (single nucleotide base change), that predicted a premature termination signal rather than the wild-type (WT) tyrosine at codon 355 (Figure 3). This mutation was absent from 5 non-FPLD3 first-degree relatives of subject II-5 (namely subjects I-2, II-2, II-4, III-1 and III-2). The mutation was absent from the genomes of 260 healthy control subjects.

### Transcriptional activity of PPARγ Y355X

**Figure 4:**
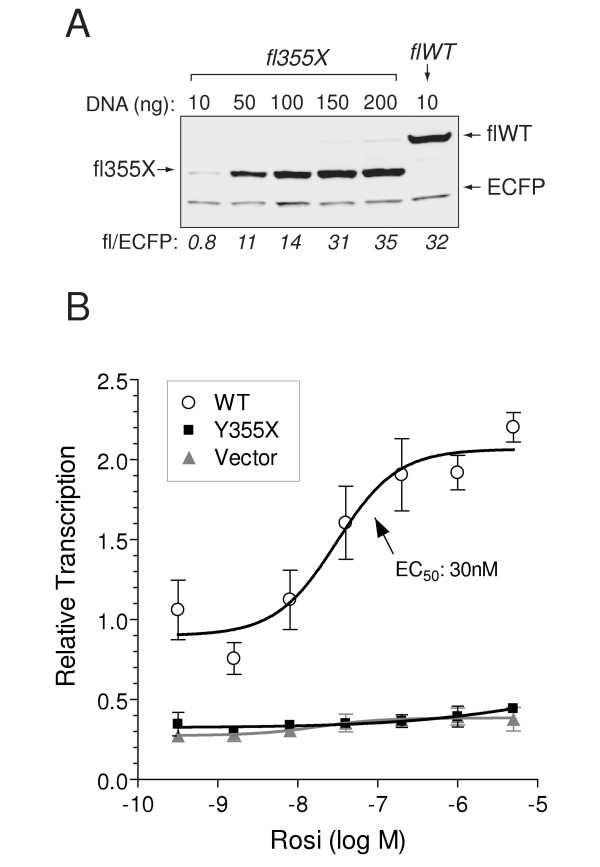
**Y355X mutation reduces PPARγ protein stability and transcriptional activity**. **(A) **Western blot of lysates from NIH 3T3 cells transfected with the indicated amount of plasmid expressing either FLAG tagged Y355X (fl355X) or wild-type (flWT) PPARγ proteins. PPARγ was visualized with anti-FLAG antibodies and ECFP produced from co-transfected plasmid (visualized with an anti-ECFP antibody) was utilized as a transfection and loading control. The numbers below the panel represent the density of each PPARγ-FLAG band normalized to the ECFP band. Roughly equal amounts of mutant and WT protein expression were achieved by a 15 fold excess of transfected mutant over WT PPARγ DNA. (**B**) Rosiglitazone dose-response curves for WT and Y355X PPARγ in NIH 3T3 cells transfected with 1 ng of WT or 15 ng of Y355X expression plasmids (non-FLAG-tagged versions) or 15 ng of empty vector, together with a PPAR responsive reporter construct (pFATP-luc) and a β-galactosidase reference plasmid. Data are normalized to the WT vehicle controls and are means ± SD (n = 3).

To establish experimental conditions that generated equal amounts of the Y355X mutation, epitope-tagged versions of the WT and mutant PPARγ clones were transfected into NIH3T3 cells. Western analysis of extracts from these cells demonstrated that much less Y355X mutant protein was produced from the same amount of transfected DNA suggesting that the mutant receptor was significantly less stable than the wild-type receptor (Figure 4A). Approximately 15 times more Y355X than wild-type DNA was needed to generate equal amounts of the two PPARγ proteins. Using these conditions, the transcriptional activity of the Y355X PPARγ mutant was assessed by transient transfection of (non-epitope tagged) PPARγ expressing plasmids into NIH3T3 cells and measuring luciferase activity from a PPAR responsive reporter. Under these conditions the Y355X mutation did not display any transcriptional activity at any dose of the ligand rosiglitazone (Figure 4B).

**Figure 5:**
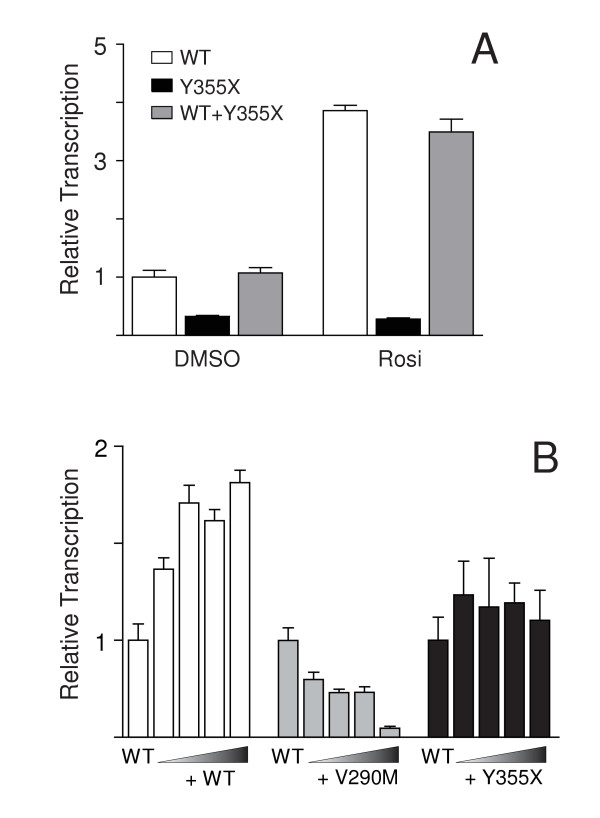
**The Y355X mutant receptor does not inhibit the activity of wild-type PPARγ**. (**A**) WT and Y355X receptors (1 and 15 ng respectively) were transfected into NIH 3T3 cells individually or in combination in the presence or absence of 5 μM rosiglitazone (Rosi). (**B**) NIH 3T3 cells were transfected with the combination of 1 ng of WT PPARγ and increasing amounts of either the WT (1–4 ng), the dominant/negative mutant V290M (1–4 ng) or the Y355X mutant receptor (1–15 ng) as indicated. Data are normalized to the transcriptional activity of 1 ng of WT receptor and are presented as means ± SD (n = 3).

To determine if the Y355X receptor had dominant-negative activity against WT PPARγ, a mixing experiment was performed in which WT and mutant receptors were co-transfected into NIH3T3 cells. In the absence of ligand, the transcriptional activity of the combination of 1 ng of WT and 15 ng of Y355X DNA (equal protein amounts) was similar to 1 ng of WT receptor alone (Figure 5A). Likewise, in the presence of a saturating amount of ligand, the presence of an equal amount of Y355X protein did not reduce the activity of co-transfected WT PPARγ. These results indicate that the Y355X mutation does not have dominant-negative activity against the WT receptor. This was confirmed by further mixing experiments in which an increasing amount of mutant or WT receptor was mixed with a fixed amount of WT PPARγ (Figure 5B). While increasing the amount of the wild-type receptor caused a dose dependent increase in transcriptional activity (Figure 5B, open bars), the addition of increasing amounts of Y355X PPARγ to a fixed amount of wild-type receptor had no significant effect on transcription (Figure 5B, filled bars). For comparison, the same experiment was conducted with a PPARγ mutation (V290M) that has been previously shown to have dominant-negative activity [6]. Increasing amounts of V290M PPARγ caused a dose-dependent decrease in wild-type PPARγ transcriptional activity in this assay (Figure 5B, grey bars). Thus, PPARγ receptors bearing the Y355X mutation do not possess any dominant-negative activity against the wild-type PPARγ receptor

## Discussion

The principal novel findings of this study are: 1) identification of a novel human *PPARG *nonsense mutation, namely Y355X; 2) demonstration that heterozygosity for Y355X is associated with partial lipodystrophy and metabolic disturbances consistent with the FPLD3 phenotype; and 3) evidence that the mechanism underlying the disease association in affected heterozygotes is haploinsufficiency rather than dominant negative interference of the function of the normal allele product. The Y355X mutation caused the complete inactivation of PPARγ transcriptional activity and reduced the stability of the PPARγ protein.

The Y355X nonsense mutation almost certainly creates haploinsufficiency – a quantitative deficiency – for PPARγ in affected FPLD3 subjects. *PPARG *Y355X (present in two FPLD3 subjects) encodes a protein that is truncated by ~30% and shows no transcriptional activity. Furthermore, the Y355X mutant receptor clearly does not display dominant-negative activity against WT receptor in transfection assays. The likelihood that this lack of dominant-negative activity reflects the in vivo situation is supported by the observation that the adult proband with Y355X had a beneficial response to an average pharmacological dose of the thiazolidinedione pioglitazone. Given the specificity of this ligand for PPARγ, this observation suggests that there is no interference of the normal allele product. The in vitro results also indicate that the Y355X mutation significantly reduces the stability of the receptor, making it likely that cells with one wild-type and one Y355X allele (as occurs in these patients), would contain significantly less mutant receptor compared to the wild-type protein. Together, these findings strongly suggest that the *PPARG *Y355X mutation acts via a haploinsufficiency mechanism.

The findings extend the range of heterozygous *PPARG *mutations seen in FPLD3 [6-10]. Currently, most heterozygous *PPARG *mutations (3/6 total heterozygous mutations, namely -14A>G, F388L and Y355X) from most affected subjects (8/12 total FPLD3 subjects), act via a haploinsufficiency mechanism. The *PPARG *-14A>G mutation within the promoter of the γ4 isoform (present in two FPLD3 subjects) was associated with quantitative loss of expression of the allele product, with no qualitative abnormalities of the encoded protein [9]. *PPARG *F388L (present in four FPLD3 subjects), a transactivation-deficient missense mutation, was expressed at normal levels and had no *in vitro *dominant negative interference with the function of the normal allele [9]. The combined LOD score for the linkage of these four heterozygous *PPARG *haploinsufficiency mutations with the FPLD3 phenotype in the three kindreds is 3.0 (maximum at 0% recombination), providing additional human genetic evidence for causation.

Only two heterozygous germline *PPARG *mutations in FPLD3, namely P467L (present in two FPLD3 subjects) and V290M (present in one FPLD3 subject), were shown by convincing *in vitro *studies to act through a dominant negative mechanism [6,7]. The *PPARG *R425C mutation (present only in one FPLD3 subject) has not yet been functionally assessed *in vitro *[10]. Another nonsense mutation in *PPARG*, namely A553ΔAAATfs185, did not show dominant negative interference of the WT allele, and carriers of this mutation did not have FPLD3, but simple insulin resistance, which required double heterozygosity with a truncation mutation in *PPP1R3A *for clinical expression. Yet another *PPARG *missense mutation, R115Q, was found in obese subjects without FPLD3; it had defective phosphorylation of serine at position 114 and an apparent gain of function resulting in accelerated differentiation of pre-adipocytes [11]. The *PPARG *mutations in subjects without FPLD3 indicate the complexity of the relationship between mutations in this gene and clinical phenotypes, such as phenotype differences related to differences in functional impact, and the requirement for interactions with other genes before a phenotype is expressed. The presence of a metabolic phenotype without loss of subcutaneous fat in the pre-pubescent carrier of Y355X suggests either some variation in penetrance of *PPARG *mutations, or some influence of other genes activated in puberty that may affect the expression of the Y355X mutation.

How might a quantitative relative loss-of-function mutation in one *PPARG *allele contribute to adipose repartitioning and insulin resistance in FPLD3? Quantitative increases in activity as induced by pharmacological PPAR agonism can enhance insulin sensitivity and anabolically influence adipose tissue [3]. But heterozygous PPARγ-deficient mice in which one allele has been deleted seem to be less insulin resistant than their WT littermates [12,13]. This suggests that an additional mechanism or stress may be required to produce insulin resistance in human homologues of these animals. A simple quantitative genetic deficiency of PPARγ might compromise the ability of heterozygotes to express sufficient PPARγ, perhaps creating a bottleneck at key stages of adipose differentiation during development and adolescence. In contrast to other direct effects of *PPARG *mutations on other pathways and targets, a simple reduction in functional activity resulting from the -14A>G, F388L and Y355X mutations may be sufficient to produce a clinical phenotype.

Interestingly, both subjects ascertained here were females. Observations from the small numbers of PPARγ-deficient individuals indicate that females may be more severely affected than males [1-3,6-10]. Similar, between-sex differences in phenotype severity were noted in FPLD2 due to mutant *LMNA *[1]. But the anecdotal nature of the between-sex differences in phenotypic severity in PPARγ-deficiency and in partial lipodystrophy precludes speculation regarding potential mechanisms. Ascertainment of additional subjects and families and specific physiological studies are required. In summary, we have found a rare nonsense mutation causing reduced expression of PPARγ in a proband with FPLD3.

## Conclusion

The findings in this Canadian kindred extend the range of *PPARG *mutations in FPLD3 and especially those that likely operate through a haploinsufficiency mechanism.

## Authors' contributions

GAF: data analysis, patient care, manuscript preparation and approval

GL: expression studies, manuscript preparation, manuscript approval

RC: patient care, manuscript approval

JW: sequencing, data analysis, editing, manuscript approval

HC: molecular studies, data analysis, editing, manuscript approval

TL: molecular studies, data analysis, manuscript preparation, manuscript approval

RAH: study design, data analysis, manuscript preparation, manuscript approval

## Pre-publication history

The pre-publication history for this paper can be accessed here:


